# SAP-expressing T peripheral helper cells identify systemic lupus erythematosus patients with lupus nephritis

**DOI:** 10.3389/fimmu.2024.1327437

**Published:** 2024-03-14

**Authors:** Yevgeniya Gartshteyn, Laura Geraldino-Pardilla, Leila Khalili, Shoiab Bukhari, Shalom Lerrer, Robert J. Winchester, Anca D. Askanase, Adam Mor

**Affiliations:** ^1^ Division of Rheumatology, Department of Medicine, Columbia University Medical Center, New York, NY, United States; ^2^ Columbia Center for Translational Immunology, Columbia University Medical Center, New York, NY, United States

**Keywords:** systemic lupus, lupus nephritis, T peripheral helper cells, T follicular helper cells, SLAM, SAP

## Abstract

**Introduction:**

T follicular (TFH) and peripheral helper (TPH) cells have been increasingly recognized as a pathogenic subset of CD4 T cells in systemic lupus erythematosus (SLE). The SLAM Associated Protein (SAP) regulates TFH and TPH function by binding to the co-stimulatory signaling lymphocyte activation molecule family (SLAMF) receptors that mediate T cell - B cell interactions. SAP and SLAMF are critical for TPH-dependent B cell maturation into autoantibody-producing plasma cells that characterize SLE pathogenesis. We hypothesized that SAP-expressing TPH cells are involved in the pathogenesis of lupus nephritis (LN).

**Methods:**

Peripheral blood mononuclear cells (PBMC) were isolated using density gradient separation from whole blood. Cells were stained for cell surface markers, followed by permeabilization and staining of intracellular SAP for spectral flow cytometry analysis. We also analyzed SAP expression from renal infiltrating LN T cells using the available single-cell RNA sequencing (scRNA seq) Accelerated Medicines Partnership (AMP) SLE dataset.

**Results:**

PBMC from 30 patients with SLE (34 ± 10 years old, 83% female), including 10 patients with LN, were analyzed. We found an increase in total SAP-positive CD4 and CD8 T cells in SLE compared with controls (55.5 ± 2.6 vs. 41.3 ± 3.4, p=0.007, and 52.5 ± 3.0 vs. 39.2 ± 2.8, p=0.007 respectively). In CD4 T cells, the highest SAP expression was in the TPH subset. The frequency of SAP^+^TPH in circulation correlated with disease activity; SLE patients with renal disease had higher levels of circulating SAP^+^TPH that remained significant after adjusting for age, sex, race, low complements, and elevated anti-dsDNA (p=0.014). scRNA-seq data of renal infiltrating T cells in LN identified SAP expression to localize to the TFH-like CD4 cluster and GZMK+ CD8 cluster. Increased SAP expression in LN was associated with the differential expression of SLAMF3 and SLAMF7 and granzyme K and EOMES. The existence of two predominant SAP-expressing subsets, the TFH-like CD4 T cells, and GZMK+ effector CD8 T cells, was verified using scRNA-seq data from a human transcriptomic atlas of fifteen major organs.

**Conclusion:**

The expansion of SAP-expressing T helper cells was associated with LN in our cohort and verified using scRNA-seq data of renal infiltrating T cells. Improved SLAM and SAP signaling understanding can identify new therapeutic targets in LN.

## Introduction

Systemic lupus erythematosus (SLE) is a heterogeneous autoimmune disease with multifactorial pathogenesis and variable organ involvement. B lymphocyte dysregulation and auto-antibody production are essential to the development of SLE. T lymphocytes help autoreactive B cells, supporting their maturation into mature B cells and autoantibody-producing plasma cells. Thus, understanding the molecular mechanisms by which CD4 T cells support B cell development is critical for understanding SLE pathogenesis.

The primary site of T-B cell differentiation is in the germinal centers of lymphoid follicles. In SLE, T-B cell interactions also occur in extra-follicular germinal centers that arise within inflamed tissues. T-B cell interactions begin following ligation of the T cell receptor (TCR) by cognate antigen presented by the B cell major histocompatibility complex (MHC). Signaling downstream of TCR-MHC binding is further modulated by secondary activating or inhibitory co-receptors that can enhance or lessen cell activation. The signaling lymphocyte activation molecule family (SLAMF) cell surface receptors, which consists of nine transmembrane proteins (SLAMF1-9), are expressed on both T and B cells and serve as co-stimulatory molecules stabilizing T-B cell interactions in the germinal centers. Genetic loci encoding the SLAMF genes are associated with SLE risk, particularly polymorphisms in SLAMF3, SLAMF4, and SLAMF6 (1–3). However, only small differences in cell surface SLAMF expression levels have been described in SLE compared to controls, which are not consistently seen across studies (4–6). We found that SLAMF3 and SLAMF6 expression can be detected on 98% of circulating T cells in SLE and healthy controls (7). Therefore, we hypothesized that the receptor’s cell-surface expression does not regulate SLAM signaling, and instead, SLAM’s role in SLE is modulated by downstream adaptors. In T cells, SLAM receptor signaling is mediated by an adaptor molecule, SLAM Associated Protein (SAP). The binding of SAP to SLAMF receptors in T cells recruits signaling kinases, leading to enhanced activation of TCR and downstream NF-kB pathways (8, 9). [The SAP gene (*SH2D1A*) is located on the X-chromosome, and the absence of SAP results in an X-linked lymphoproliferative disease, XLP, an immunodeficiency syndrome characterized by unstable T-B cell interactions, absence of germinal centers, and lack of isotype switched B cells (10). On the other hand, a loss of function SAP frameshift mutation in mouse models of SLE protected the mice from developing lupus, specifically the production of autoantibodies and the development of glomerulonephritis (11).

T follicular helper cells (TFH), a subset of the CD4 T helper cells, function in the secondary lymphoid organs to promote B cell differentiation. Recognized as PD-1^+^CXCR5^+^CD4^+^ T cells, TFH are recruited to the lymphoid follicles, where they promote B cell maturation through direct T-B cell contact via co-stimulatory receptors and cytokine secretion. T peripheral helper cells (TPH) are a more recently identified subset of CD4 T cells that are transcriptionally and functionally similar to TFH. TPH cells, defined as PD-1^HIGH^CXCR5^-^CD4^+^, were first isolated from the joints of patients with rheumatoid arthritis and have since been shown to be expanded in lupus and other autoimmune conditions (12–14). TPH maintain the ability to support B cell development and antibody production. Still, instead of localizing to the secondary lymphoid tissues, these cells can be isolated from the blood and peripheral tissues, especially at sites of inflammation.

SAP deficiency results in unstable T-B cell interactions and the absence of antigen-specific antibodies (15, 16). In contrast, SLE is a disease of increased ectopic germinal center formations and class-switched, long-lived B cells that reflect stable TFH-B cell interactions. This led us to hypothesize that SLAMF6/SAP signaling, especially in the TFH and TPH cells, is enhanced in SLE and contributes to its pathogenesis.

## Material and methods

### Patients

The study population was a sample of 30 adult patients randomly recruited from the Columbia University Lupus Cohort. All patients met the 1997 American College of Rheumatology (ACR) classification criteria. Demographic information was obtained from chart review. Serological covariates were measured at the New York Presbyterian Hospital clinical laboratory and ascertained from chart review; blood specimens for clinical and research use were drawn simultaneously. The Columbia University Institutional Review Board approved the study.

Eighty-three percent were female; the mean age was 34 ± 10 (**Table 1**). Race was reported by patient self-report. Ten percent of the patients were White, 27% were Black, and 63% were Hispanic. According to New York City Department of Health neighborhood population estimates (modified from US Census Bureau population estimates), the racial/ethnic distribution of the Manhattan population in 2022 was 31.9% non-Hispanic white, 28.9% Hispanic, 23.4% non-Hispanic black, 14.2% non-Hispanic Asian and 1.6% other. However, the area around Columbia University is known to have a more significant percentage of people of Hispanic ethnicity and African descent. For these reasons, despite the random recruitment of sequential consenting patients, our study population has more minority groups than population data alone would predict.

**Table 1:** Patient Characteristics.

	SLE (all patients)			HC (n=13)
	no LN (n=20)	LN (n=10)	p-value	
Age	35.9 ± 11.4	31.2 ± 6.9	0.24	30.3± 6.2
Sex (Female)	19 (95%)	6 (60%)	**0.03**	10 (78%)
Race				
White	2 (10%)	1 (10%)	1.00	6 (46%)
Black	6 (30%)	2 (20%)	0.68	2 (15%)
Hispanic	12 (60%)	7 (70%)	0.70	2 (15%)
Other				3 (23%)
Weight (kg)	75.0 ± 21.4	83.3 ± 35	0.71	–
Ever smoker	2 (10%)	2 (20%)	0.58	–
Disease duration (years)	3[1-11]	10.5[4-15]	0.26	–
non-renal SLEDAI-2K	6[3-8]	5[2-8]	0.59	–
ACR criteria				
malar rash	11 (55%)	7 (70%)	0.69	–
discoid rash	6 (30%)	3 (30%)	1.00	–
photosensitivity	10 (50%)	2 (20%)	0.24	–
mucositis	8 (40%)	5 (50%)	0.71	–
arthritis	17 (85%)	10 (100%)	0.53	–
serositis	9 (45%)	4 (40%)	1.00	–
hematological	7 (35%)	6 (60%)	0.26	–
Serologies				
ANA	20 (100%)	10 (100%)	–	–
anti-dsDNA	17 (85%)	9 (90%)	0.60	–
anti-Smith	7 (35%)	5 (50%)	0.46	–
aPL	8 (40%)	3 (33%)	1.00	–
Medication				
HCQ	11 (55%)	6 (60%)	0.55	–
Mycophenolate mofetil	4 (20%)	5 (50%)	0.11	–
Calcineurin inhibitors	0	2 (20%)	0.10	–
Biologics (anti-B cell)	1 (5%)	1 (1%)	0.56	–
Cyclophosphamide	0	1 (1%)	0.33	–
Prednisone (>10mg/day)	5 (25%)	4 (40%)	0.33	–

aPL, antiphospholipid antibodies; HCQ, hydroxychloroquine; HC, healthy control. Values in bold indicate p<0.05.

Clinical lupus disease activity was measured using the SLEDAI-2000 (SLEDAI-2K) (17). The definition of a lupus flare was based on the SELENA-SLEDAI Flare Index (18). Ten patients had kidney biopsies confirmed lupus nephritis (LN). The median time from kidney biopsy to study enrollment was 228 days (IQR 5-1983 days). Class IV and Class V were present in 2 and 3 patients, respectively; the remaining five patients had an overlap of class IV/V LN. Of the ten patients with LN, nine had active renal disease at time of specimen collection. Active renal disease was characterized by proteinuria with urine protein creatinine ratio (UPCR)>0.5g/g (9 patients), pyuria (4 patients), hematuria (3 patients), low complements (7 patients) and elevated anti-double stranded DNA (dsDNA) antibody level (6 patients). History of biopsy proven LN was associated with male sex (p=0.02) and conferred increased disease activity, as demonstrated by higher SLEDAI-2K scores.

### General reagents

RPMI medium 1640, PBS, and FBS were purchased from Life Technologies. Staphylococcus Enterotoxin E (SEE) was acquired from Toxin Technology. All stimulations were performed with purified anti-human CD3 (BioLegend #300465), anti-human SLAMF6 (BioLegend #317202) and anti-human SLAMF3 (BioLegend #326109). Anti-SAP monoclonal antibodies were purchased from eBioscience (#50-138-27).

### Cell isolation

Peripheral blood mononuclear cells (PBMC) from SLE patients and healthy controls were isolated from peripheral blood using Ficoll gradient centrifugation. Red blood cells were lysed using a lysis buffer (Gibco #A1049201). Primary CD3^+^ T and B cells were isolated using RosetteSep T cell enrichment kit (STEMCELL #15021C) and B cell enrichment kit (STEMCELL #15024C), respectively. All cells were stored at -80C until analysis.

### Cell stimulation

For immobilization, plate-bound stimulation, 48 well plates were coated with anti-CD3 1.5ug/mL and either anti-SLAMF6, anti-SLAMF3, or inactive IgG isotype control at a concentration of 5ug/mL. PBMCs were incubated for 12-18 hours at a density of 500,000 cells per well. For soluble stimulation, primary autologous T and B cells were co-cultured in a 2:1 ratio in the presence of SEE 150pg/mL. anti-SLAMF6 or anti-SLAMF3 antibodies were added to the co-cultures at a concentration of 10ug/mL.

### Spectral flow cytometry analysis

Viability was assessed using Zombie UV dye (BioLegend #423107) and nonspecific Fc interactions were blocked (BioLegend #422302). Cells were stained for cell surface markers as follows: APC/Cy7 anti-CD3 (BioLegend #300318), AF700 anti-CD4 (BioLegend #300526), BV605 anti-CD8a (BioLegend #301040), BV421 anti-PD1 (BioLegend #329920), PE/Cy7 anti-CXCR5 (BioLegend #356924), BV711 anti-CD127 (BioLegend #351328), PE/Cy5 anti-CD25 (BioLegend #302608), PE anti-HLA-DR (BioLegend #307606) and BV510 anti-CD69 (BioLegend #310935). Brilliant Stain Buffer was obtained from BD Biosciences (#563794). Following staining, cells were fixed (BioLegend #420801) and permeabilized (BioLegend #421002) followed by intracellular SAP staining (eBioscience #50-9787-42). Samples were analyzed in five batches containing SLE and healthy control samples. A single reference control sample from the same donor was analyzed in each set to ensure internal validity. Data was acquired on the 5-Laser Cytek Aurora and analyzed using FlowJo v.10.9. Gating strategy is provided in the supplement. (**Supplementary Figure 1**).

### Confocal microscopy

SLAMF6-GFP fusion expression constructs were generated by cloning SLAMF6 (DNASU # HsCD00446754) into a pEGFP-N1 vector (Invitrogen). DNA expression constructs were introduced into Jurkat T cells by nucleofection according to the recommended protocol (Lonza Nucleofector II). Raji B cells were prestained with CellTrace Violet (Invitrogen #C34557, 1:1,000 dilution for 20 min in PBS). In some conditions, Jurkat T cells were pretreated with either anti-SLAMF6 or anti-SLAMF3; Raji B cells were coated with 2mg/mL SEE. Next, 2x10^5^ Jurkat T cells were mixed with 2x10^5^ Raji B cells; the co-culture was placed on a glass bottom culture dish (MatTek Corporation) and rested for 60 minutes to allow conjugates to form. Confocal images were acquired on a Zeiss LSM 900 confocal microscope and analyzed with ZEN Blue software.

### Sequencing data analysis

We used the single-cell RNA sequencing (scRNA-seq) data from SLE kidney biopsy samples, publicly available through the Accelerated Medicines Partnership (AMP) national collaboration (accession number SDY997) (19). Briefly, leukocytes isolated from kidney biopsies from 24 patients with LN and ten control samples, acquired from living donor kidney biopsies, were analyzed by scRNA-seq and subjected to stepwise cell clustering to identify cell-specific populations within the kidney. We accessed the scRNA-seq data using the Bioturing Talk2Data platform. Using the original authors’ annotation of the clusters, we compared the expression of the SAP gene, *SH2D1A*, in T cells from SLE vs. control biopsy samples.

Additionally, we accessed scRNA-seq data sequenced from fifteen major organs of a single healthy volunteer (accession number GSE159929) (20). Briefly, high-throughput sequencing was performed on viable single cells from the tissue samples of 15 different organs of a research-consented adult donor, ultimately achieving an average of 6,000 cells for each organ. We restricted our analysis to T cells and used normalized gene counts as reported by the Bioturing platform. Clustering was performed using the Louvain method. Visualization was performed using the Uniform Manifold Approximation and Projection (UMAP) algorithm.

### Statistical analysis

Continuous variables were summarized as mean ± standard deviation for normally distributed variables or median ± interquartile range (IQR) for non-normally distributed variables and analyzed using a Student’s t-test or Man-Whitney U respectively. Categorical variables were summarized as frequencies and analyzed using Fischer Exact test. Multivariable modeling was done in Stat/IC 15.1.

Differential gene expression was calculated using the T-test statistic method, with log2FC ≥ 0.6 and a false discovery rate (FDR) ≥ 0.05. Data was plotted using the ggvolcano shiny app. Pathway analyses of enriched genes were assessed using the Kyoto Encyclopedia of Genes and Genomes (KEGG) and Gene Ontology (GO) resources.

## Results

### SAP levels are increased in T cells from SLE patients

**Figure 1:**
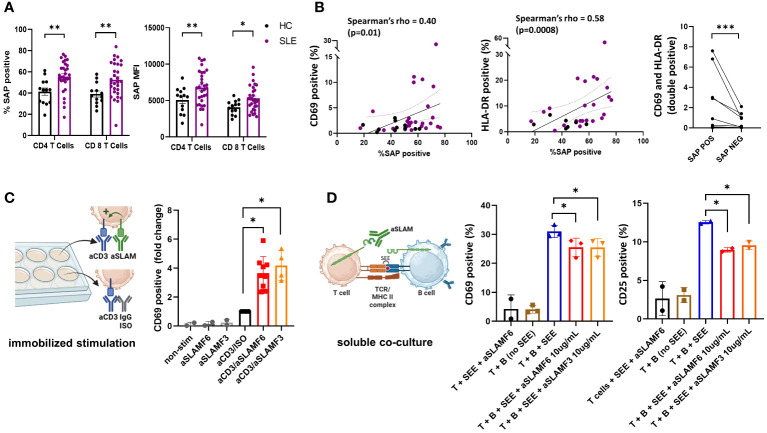
SAP levels are increased in T cells isolated from SLE patients and function to enhance T cell activation. **(A)** Peripheral blood mononuclear cells (PBMC) from SLE patients (n=30) and healthy controls (n=13) were evaluated by flow cytometry for SAP expression in T cells, summarized as percent of SAP positive T cells (%SAP positive) or median SAP MFI per person. **(B)** Levels of cell surface CD69 (left) and HLA-DR (center) expression on circulating CD4 T cells were quantified and plotted against SAP expression levels, each dot represents a study subject. Frequency of T cells double positive for CD69 and HLA-DR by SAP expression are shown (right). Each spaghetti plot represents a study subject. **(C)** PBMCs isolated from SLE patients were stimulated *in-vitro* (500,000 cells per well, in a 48 well plate) for 4-6 hours with either anti-CD3 (aCD3) alone or aCD3 and anti-SLAMF6 (aSLAMF6) or anti-SLAMF3 (aSLAMF3) (plate-bound stimulation). Following stimulation cells were collected and assessed for CD69 cell surface expression by flow cytometry. **(D)** Primary autologous T and B cells isolated from healthy controls were co-cultured for 12-16 hours in presence of staphylococcal superantigen (SEE, 150pg/mL) (soluble co-culture). aSLAMF6 or aSLAMF3 antibodies were added to the co-cocultures to disrupt SLAMF receptor binding in the T-B immunological synapse. Cells were then collected and assessed for CD69 and CD25 cell surface expression. The statistical comparisons in this figure were done using a two-sided, unpaired t-test. *p<0.05, **p<0.01, ***p<0.001. The images were created with BioRender.com.

Using spectral flow cytometry, we analyzed the intracellular expression of SAP in circulating, non-stimulated T cells. We found an increase in total SAP-positive CD4 and CD8 T cells in SLE as compared with controls (55.5 ± 2.6 vs. 41.3 ± 3.4, p=0.007 and 52.5 ± 3.0 vs. 39.2 ± 2.8, p=0.007 respectively). Similarly, SAP staining intensity was more significant in CD4 and CD8 T cells in SLE (MFI 6750.8 ± 423.3 vs. 5065.9 ± 488.6, p=0.02 and 5301.8 ± 311.6 vs. 4069.9 ± 265.1, p=0.05 respectively) (**Figure 1A**). We validated the increased SAP level in T cells from SLE patients (blood and synovial fluid), as compared to healthy controls using a western blot analysis (**Supplementary Figure 2**). To better understand whether SAP expression level was associated with an activated T cell phenotype, we looked at the association between SAP levels and HLA-DR and CD69 expression in circulating T cells (**Figure 1B**). We found a positive correlation between SAP expression and HLA-DR (Spearman’s rho=0.58, p<0.001) as well as between SAP levels and CD69 (Spearman’s rho=0.40, p=0.01). Frequency of circulating T cells double positive for CD69 and HLA-DR was higher in SAP positive as compared to SAP negative T cells. We therefore identified that a higher percentage of peripheral T cells from SLE patients, compared to healthy controls, express SAP and that these circulating T cells further express markers suggesting recent activation.

To better understand the functional role of SAP in SLE, we focused on the SLAMF receptor signaling pathways dependent on SAP binding. Given that polymorphisms in SLAMF3 and SLAMF6 are associated with SLE risk, we chose to focus on these two SLAMF receptors for our functional experiments. SAP is a SLAM adaptor protein with two ITSM binding sites, one of which binds to SLAM family receptors, and the other recruits signaling kinases, ultimately coupling SLAM signaling to TCR activation (9). SLAMF receptors can have an activating or a inhibitory effect on the TCR depending on presence or absence of intracellular SAP, respectively (8). We have previously shown that in healthy T cells, signaling through SLAMF6 receptors enhanced T cell activation (21). We hypothesized that in SLE, SLAMF6 should function similarly to enhance T cell activation. To confirm T cell co-stimulation through SLAMF6 and SLAMF3 in SLE, we stimulated PBMCs isolated from SLE patients with anti-CD3 antibodies alone or in the presence of activating anti-SLAMF6 or anti-SLAMF3 antibodies (immobilized stimulation, **Figure 1C**). We found that T cells activated with anti-CD3 + anti-SLAMF6 or anti-CD3 + anti-SLAMF3 expressed higher levels of CD69 than T cells activated with anti-CD3 antibody alone.

Similarly, we would expect that in circulation homotypic binding of SLAMF receptors expressed by both T and B cells (i.e., SLAMF6 or SLAMF3) supports cell-cell interaction and enhances MHC-TCR activation. We thus asked whether anti-SLAMF6 or anti-SLAMF3 antibodies added to a T-B cell co-culture can disrupt T-B cell SLAMF-SLAMF binding and thus dampen T cell activation (soluble stimulation, **Figure 1D**). We used confocal microscopy to confirm the ability of anti-SLAMF6 antibodies to disrupt SLAMF6 enrichment in the immunological synapse contact zone formed during T-B cell interaction. (**Supplementary Figure 3**). Indeed, we found that adding either anti-SLAMF6 or anti-SLAMF3 antibodies to an autologous T-B cell co-culture inhibited the expression of CD69 and CD25 on T cells. We thus show that inhibition of SLAMF receptors with competitive monoclonal antibody binding can weaken T-B cell interactions.

### SAP^+^ TPH levels are expanded in SLE and are associated with renal involvement

**Figure 2:**
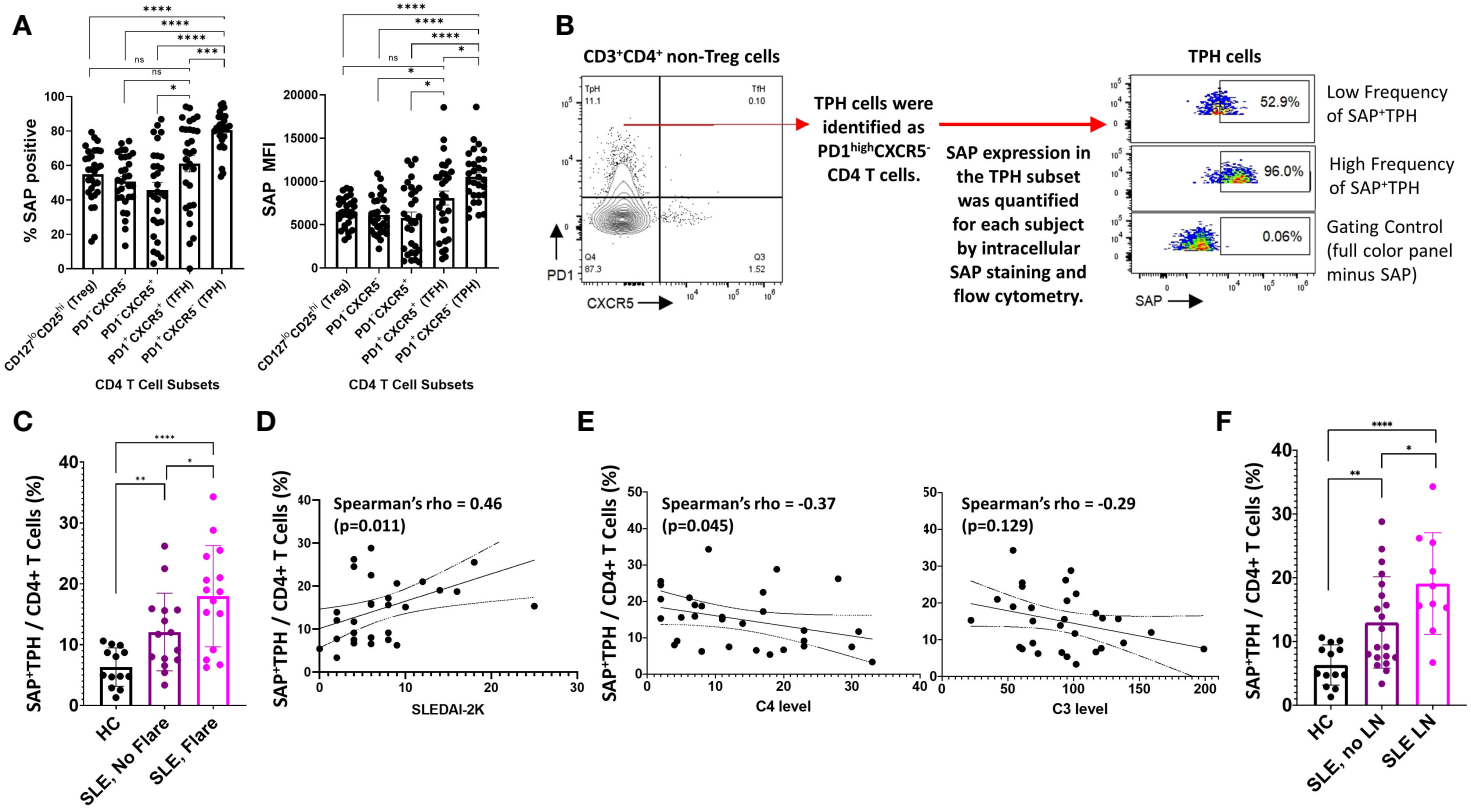
SAP^+^TPH cells are expanded in SLE, particularly in biopsy-proven lupus nephritis (LN). **(A)** PBMC samples from SLE patients (n=30) were analyzed by flow cytometry to identify CD4 and CD8 T cell subsets in circulation. In the CD4 T cell subsets, T regulator cells (Treg) were gated out and the remaining CD4 T cells were further sub-divided based on PD1 and CXCR5 expression. The average frequency of SAP expression and median SAP MFI were summarized by CD4 subsets. **(B)** TPH cells, defined as PD1^high^CXCR5^-^ were further analyzed based on SAP expression, identifying a new subset of SAP expressing TPH cells (SAP^+^TPH). **(C)** The frequency of SAP^+^TPH cells in healthy controls (n=13), stable SLE (n=15), and SLE with disease flare (n=15) was quantified and compared. SLE flare was defined based on the SELENA-SLEDAI Flare Index (18). **(D)** We evaluated for a correlation between individual SAP^+^TPH levels and SLEDAI-2K clinical disease activity scores and between **(E)** SAP^+^TPH and peripheral blood complement C4 and complement C3 levels (mg/dL). Statistical correlation was assessed using the Spearman’s rho. **(F)** The frequency of SAP^+^TPH cells in healthy controls (n=13), non-renal SLE (n=20) and SLE with LN (n=10, 9 patients with active LN and 1 patient in clinical remission) was quantified and compared. The statistical comparisons in this figure were done using a two-sided, unpaired t-test. *p<0.05, **p<0.01, ***p<0.001, ****p<0.0001. ns, non-significant.

SAP expression in T cells is critical for T cell-mediated B cell maturation (10, 16). This led us to evaluate the expression of SAP in CD3^+^CD4^+^PD1^+^CXCR5^+^ TFH cells and the CD3^+^CD4^+^PD1^+^CXCR5^-^ TPH cells. In the SLE donors, TFH cells and TPH cells constituted 2.3 ± 2.8% and 18.7 ± 9.7% of the CD4 T cells. TPH cells were the main producers of IL21 at rest, and consistently upregulated IL21 production following stimulation (**Supplementary Figures 4A, B**). Akin to the initial description of TPH cells in RA not producing IL2 following stimulation, we found that it was the synthesis of IL21, rather than IL2, that increased following stimulation of SLE TPH cells (**Supplementary Figure 4C**) (12). We found that the number of T cells with detectable SAP levels, as well as the median SAP expression level, was significantly greater in TPH population (80%, MFI 10537) as compared to either the PD1^-^CXCR5^-^ T cell subset or the CD127^lo^CD25^hi^ T regulatory cell subset (51%, MFI 6128 and 55%, MFI 6486 respectively, p<0.0001). On the other hand, we found considerable variability across subjects in SAP expression levels in TFH cells, with no significant difference seen in the TFH population as compared to the PD1^-^CXCR5^-^ or the T regulatory cells (**Figure 2A**). Thus, we found that SAP expression is not uniform across all T cells and that the TPH subset is particularly enriched in SAP expression.

Given the correlation of SAP expression with markers of recent T cell activation, we hypothesized that SAP-positive TPH may identify a subset of TPH cells that more directly contribute to the dysregulated humoral immunity and clinical outcomes in SLE. We defined these cells as SAP-positive TPH or SAP^+^ TPH (**Figure 2B**). SAP^+^ TPH cells constituted 6.3 ± 3.0% of all CD4 T cells in the healthy controls and were expanded to 11.8 ± 6.3% in SLE patients with stable disease (15 patients, p=0.006) and 18.8 ± 8.0% in SLE patients experiencing a flare (15 patients, p<0.0001 compared to healthy controls). SAP^+^ TPH levels were significantly greater in SLE patients’ experiencing a flare as compared to SLE patients with stable disease (p=0.04) (**Figure 2C**). In SLE, SAP^+^ TPH levels correlated with SLEDAI-2K scores (**Figure 2D**) and showed an inverse correlation with complement C4 levels (**Figure 2E**).

Given the heterogeneity of SLE disease flares, we explored the association between specific disease activity domains present at visit and the level of SAP^+^ TPH cells in circulation. We found that an expansion of SAP^+^ TPH cells was associated with features of LN, such as the presence of nephrotic syndrome and an active urine sediment (defined by >5 RBCs or WBCs per high power field on urine microscopy) on the SLEDAI-2K (**Table 2**).

**Table 2:** Association of SAP^+^TPH levels with SLE SLEDAI-2K Disease Activity Domains.

SLE Disease Activity Domains:	Association with %SAP^+^TpH p-value
Arthritis (n=14)	0.62
Mucocutaneous (n=9)	0.98
**Lupus nephritis (n=10):**	**0.05**
Proteinuria (>0.5g/day) (n=9)	0.15
**Urine Sediment (n=4)**	**0.05**
**Nephrotic syndrome (n=5)**	**0.02**
Alopecia (n=5)	0.62
Cardiorespiratory:	
Pleural effusions (n=1)	0.18
Pericarditis (n=1)	0.09
Cytopenia (n=4)	0.90
Low complements (n=16)	0.34
**Increased anti-dsDNA (n=19)**	**0.07**

Values in bold indicate p<0.05.

In a univariable analysis, SAP^+^ TPH levels were significantly greater in the SLE patients with a history of biopsy confirmed LN as compared with SLE patients without renal involvement (19.1 ± 8.0 vs. 13.0 ± 7.2, p=0.04) (**Figure 2F**). Importantly, TPH levels without SAP quantification did not differentiate renal from non-renal SLE involvement (23.0 ± 10.2 vs. 16.7 ± 9.0, p=0.10). We analyzed the data using a multivariable model that included age, sex, race, low complement levels and elevated anti-dsDNA antibody levels as potential confounders in the association of SAP^+^ TPH cells with LN. Controlling for these confounders, the level of SAP^+^ TPH cells remained significantly associated with occurrence of LN, such that for every one-point increase in the percent of circulating SAP^+^ TPH cells there was a 3% increase in the odds of having LN (p=0.014) (**Table 3**).

**Table 3:** Multivariable analysis of clinical variables associated with lupus nephritis.

Lupus Nephritis (LN):	β Coeff	Std Error	p-value
Age	-0.01	0.01	0.144
Male sex	0.58	0.20	**0.010**
Race	-0.01	0.12	0.936
SAP^+^TPH/CD4 T Cells (%)	0.03	0.01	**0.014**
Low complement (C3 or C4)	0.09	0.15	0.562
Increased anti-dsDNA	-0.03	0.23	0.882

Values in bold indicate p<0.05.

**Figure 3:**
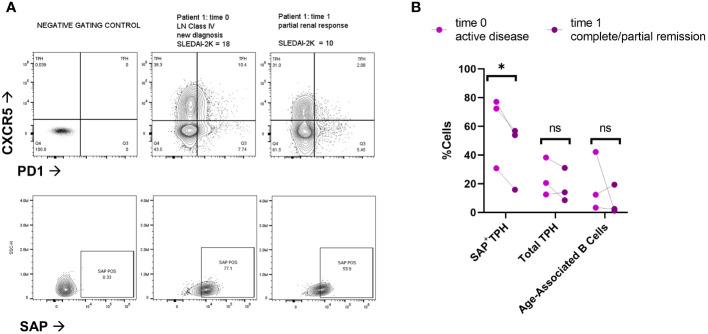
SAP positive TPH cells decrease following treatment of active lupus disease. PBMC samples were collected from three patients before (time 0) and 6-9 months after (time 1) treatment with mycophenolate mofetil (2-3 grams/day), initiated for active systemic lupus. Disease activity at each time point was measured with the SLEDAI-2K (17). **(A)** PBMC were analyzed for PD1, CXCR5 and SAP expression using spectral flow cytometry. Representative plots are shown from two patients with lupus nephritis before and after treatment initiation. Partial renal response was defined as stabilization of serum creatinine and a >50% decrease in proteinuria. **(B)** Quantification of SAP^+^TPH, total TPH cells, and Age-Associated B cells (CD19+, CD21-low, CD11c+). The statistical comparison was done using a paired t-test. *p<0.05. ns, non-significant. FMO, full-color panel minus one color (negative gating control); t, time; LN, lupus nephritis.

To evaluate for a direct association between SAP expression and clinical response, we analyzed paired samples from three SLE patients at the time of active disease (time=0) and 6-9 months after treatment initiation (**Figure 3**). Two of the three patients had active class IV lupus nephritis, treated with mycophenolate mofetil; the third patient had a myositis and arthritis flare and was also treated with mycophenolate mofetil. At time of treatment initiation, t=0, the median SLEDAI-2K was 18 [9-18]. Following treatment, t=1, the median SLEDAI-2K was 4 [0-10] with all patients achieving either partial or complete remission. We found that clinical improvement was associated with decreased circulating SAP^+^ TPH cells in each of the three patients. On the other hand, neither the total TPH cells nor the pathogenic B cell subsets (CD21^low^CD11c^+^) showed a consistent decline with clinical improvement.

**Figure 4:**
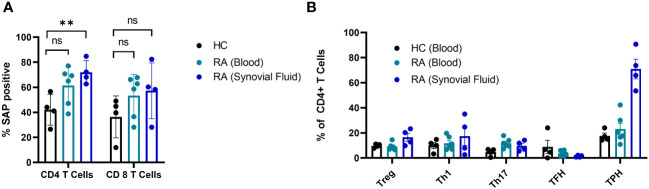
SAP levels are increased in T cells isolated from synovial fluid in patients with rheumatoid arthritis. **(A)** Peripheral blood mononuclear cells (PBMC) (n=6) and synovial fluid mononuclear cells from the same patients (n=4), as well as healthy controls (n=4) were evaluated by flow cytometry for SAP expression in T cells, summarized as percent of SAP positive T cells (%SAP positive). **(B)** CD4 T cell subsets were analyzed in the blood and synovial fluid of healthy controls and patients with rheumatoid arthritis. Treg, T regulatory cells; Th1, T helper 1 cells; Th17, T helper 17 cells. The statistical comparisons were done using a two-sided, unpaired t-test. **p<0.01. ns, non-significant.

In summary, SAP^+^ TPH cells were expanded in the circulation of patients with active SLE, especially LN. Furthermore, a decrease in this CD4 T cell subset was associated with clinical response following treatment initiation. To validate the existence of this subset in other autoimmune conditions, we measured SAP expression in T cells from peripheral blood and matched synovial fluid taken from patients with rheumatoid arthritis (RA). We found a significant expansion of SAP^+^ CD4 T cells in the synovial fluid, and a trend for increased SAP^+^ CD4 cells in the circulation of RA patients as compared to healthy control (**Figure 4A**). On further analysis of the synovial fluid, we found that 71 ± 8% of the CD4 T cells in the RA joint were comprised of TPH cells (**Figure 4B**). Thus, the SAP^+^ TPH likely traffics from the peripheral circulation to the inflamed tissue site, such as the RA joint, where it is the predominant helper T cell phenotype mediating the local inflammatory response.

### SAP expression localizes to TFH-like CD4 and GZMK+ CD8 kidney infiltrating T cells in LN

**Figure 5:**
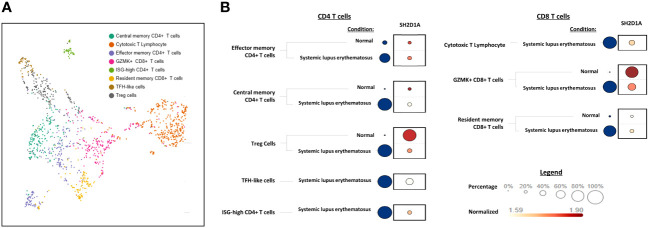
SAP expression localizes to TFH-like CD4 and GZMK+ CD8 kidney infiltrating T cells in LN. Single cell RNA sequencing (scRNA-seq) data from SLE kidney biopsy samples, publicly available through the Accelerated Medicines Partnership (AMP) national collaboration, was analyzed for normalized SAP gene (SH2D1A) expression. **(A)** Dimension reduction of 1,334 T cells was performed using the Uniform Manifold Approximation and Projection (UMAP) algorithm. Cell clusters are shown according to the original cell annotation by the AMP authors (19). **(B)** A dot plot of *SH2D1A* expression by T cell cluster. Blue circles encode the percentage of cells from each condition (Systemic lupus erythematosus vs. Normal) that make-up the T cell cluster. The larger dot diameter indicates a greater proportion of cluster cells originating from the condition specified. *SH2D1A* expression is encoded by dots colored on a yellow-red scale to indicate average gene expression from the cells in which the gene was detected. The larger dot diameter indicates that the gene was detected in a greater proportion of cells from the cluster. CD4 T cells are shown on the left and CD8 T cells are shown on the right.

Based on our finding of the association between SAP^+^ TPH and LN, we hypothesized that this SAP^+^ subset of T cells traffics to the kidney, akin to what we found in the RA joint. We used scRNA-seq data from SLE kidney biopsy samples, available through the AMP collaboration, to validate these findings in a LN cohort (19). UMAP analysis was used to visualize the kidney-infiltrating T cells based on the original authors’ annotation of the clusters (**Figure 5A**). We quantified the expression of the SAP gene, *SH2D1A*, across these T cell clusters from SLE vs. control biopsy samples (**Figure 5B**). SAP expression from healthy kidney CD4 T cells was predominantly seen in T-regulatory cells. In contrast, in SLE kidney CD4 T cells, SAP expression localized to the “TFH-like T cell” cluster, the latter annotated based on high expression of CXCR5, PD1, CXCL13, and MAF transcription factor (**Figure 5B** left). In the CD8 T cells, SAP expression localized to the cytotoxic and granzyme K (GZMK) expressing CD8 T cell populations that were detected in higher proportion in LN as compared to control kidney (**Figure 5B** right). In summary, SAP expression in the LN kidney was predominantly localized to TFH-like CD4 T cells as well as the GZMK-expressing CD8 T cells.

**Figure 6:**
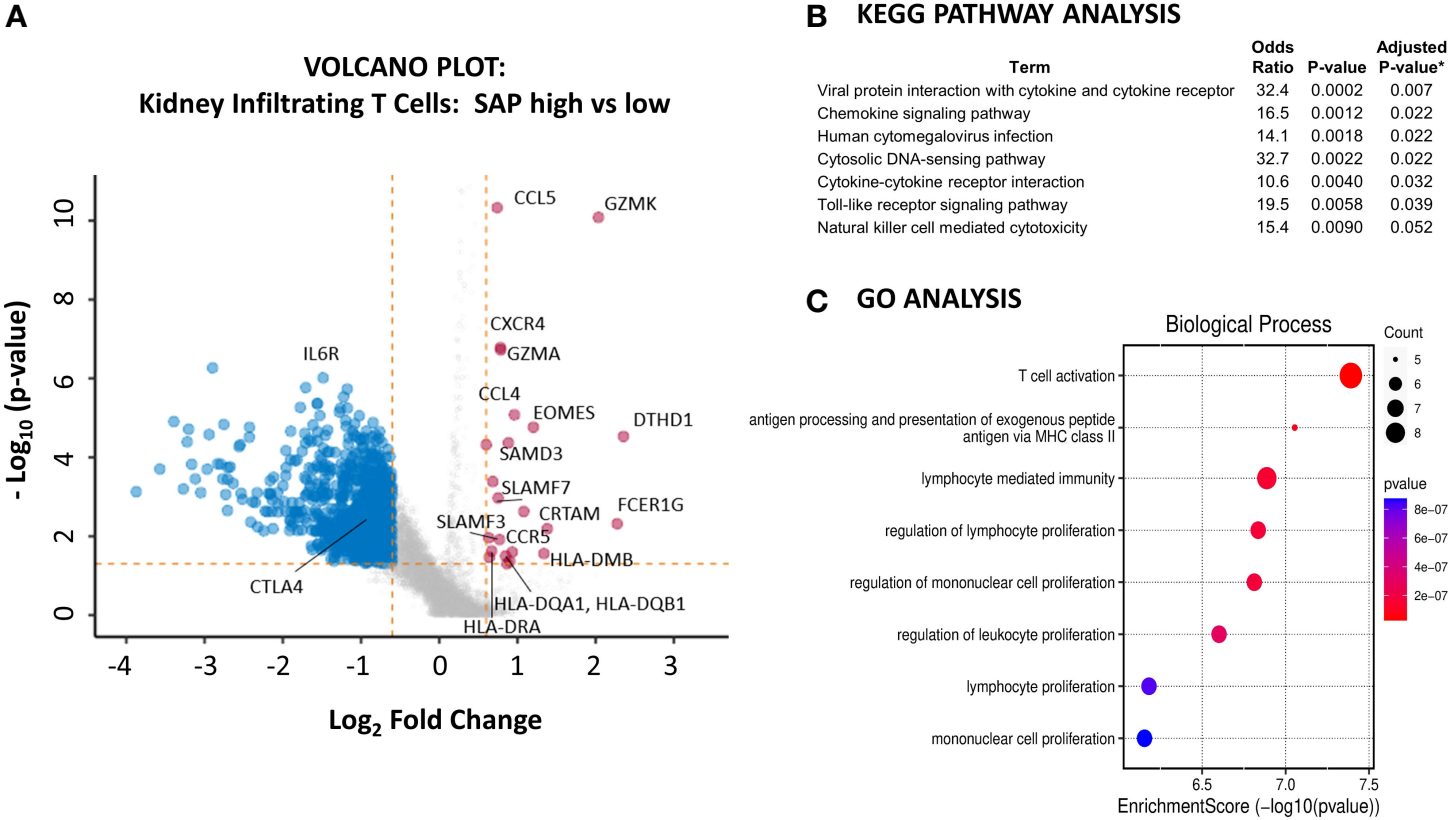
Kidney infiltrating T cells that express high levels of SAP have upregulated expression of genes involved in pro-inflammatory T cell activation. **(A)** A volcano plot was generated by comparing the differential gene expression of SAP-high as compared with SAP-low kidney infiltrating T cells from LN biopsy samples. Control biopsy samples were not included in this analysis. SAP-high T cells were selected based on the top quartile of normalized SAP gene expression. SAP-low T cells were selected based on the lowest quartile of normalized SAP gene expression. The volcano plot shows upregulated and downregulated genes (log2FC ≥ 0.6 and a false discovery rate (FDR) ≥ 0.05). **(B)** KEGG Pathway and **(C)** GO Analysis of upregulated genes associated with SAP expression in kidney infiltrating T cells. *Adjusted p-value computed using the Benjamini-Hochberg method of correction for multiple comparisons.

We next sought to understand better the transcriptomic signature of LN kidney T cells expressing high as compared to low levels of SAP. We used the normalized SAP gene expression levels to identify the differentially expressed genes between the highest and lowest quartiles of SAP-expressing T cells (**Figure 6A**). Increased SAP expression was associated with the expression of *SLAMF3* and *SLAMF7* and class II antigen-presentation MHC molecules. At the same time, differential upregulation of *GZMK, GZMA* and *EOMES*, *CRTAM*, and *DTHD1* all suggested an activated, effector CD8 presence. Finally, upregulated *CCL4* (MIP-1B), *CCL5* (RANTES), *CXCR4*, and *CCR5* cytokine gene expression were also noted. Interestingly, the *IL6R* was downregulated in SAP-high T cells. We next analyzed the enriched gene set using KEGG and GO pathway analyses to understand better the functions associated with high SAP expression in T-cells. KEGG analysis identified anti-viral responses, chemokine signaling, cytosolic DNA-sensing cytokine interaction, and Toll-like receptor signaling pathways (**Figure 6B**). GO analysis identified enrichment in biological processes associated with T cell activation, MHC Class II peptide presentation, and lymphocyte-mediated immunity (**Figure 6C**). These results confirmed the presence of SAP expressing “TFH-like” cells in the kidneys of LN that was not seen in the control kidney. Additionally, in CD8 T cells, SAP gene expression was seen in the *EOMES* ^+^ and *GZMK* ^+^ T cells expressing pro-inflammatory cytokine and chemokine mediators akin to what is commonly seen in anti-viral T-cell activation responses.

### SAP expression localizes to TFH/TPH-like CD4 T cells and *GZMK* ^+^ *CCL4* ^+^ *CCL5*+ CD8 T cells in an adult human transcriptome of major systemic organs

**Figure 7:**
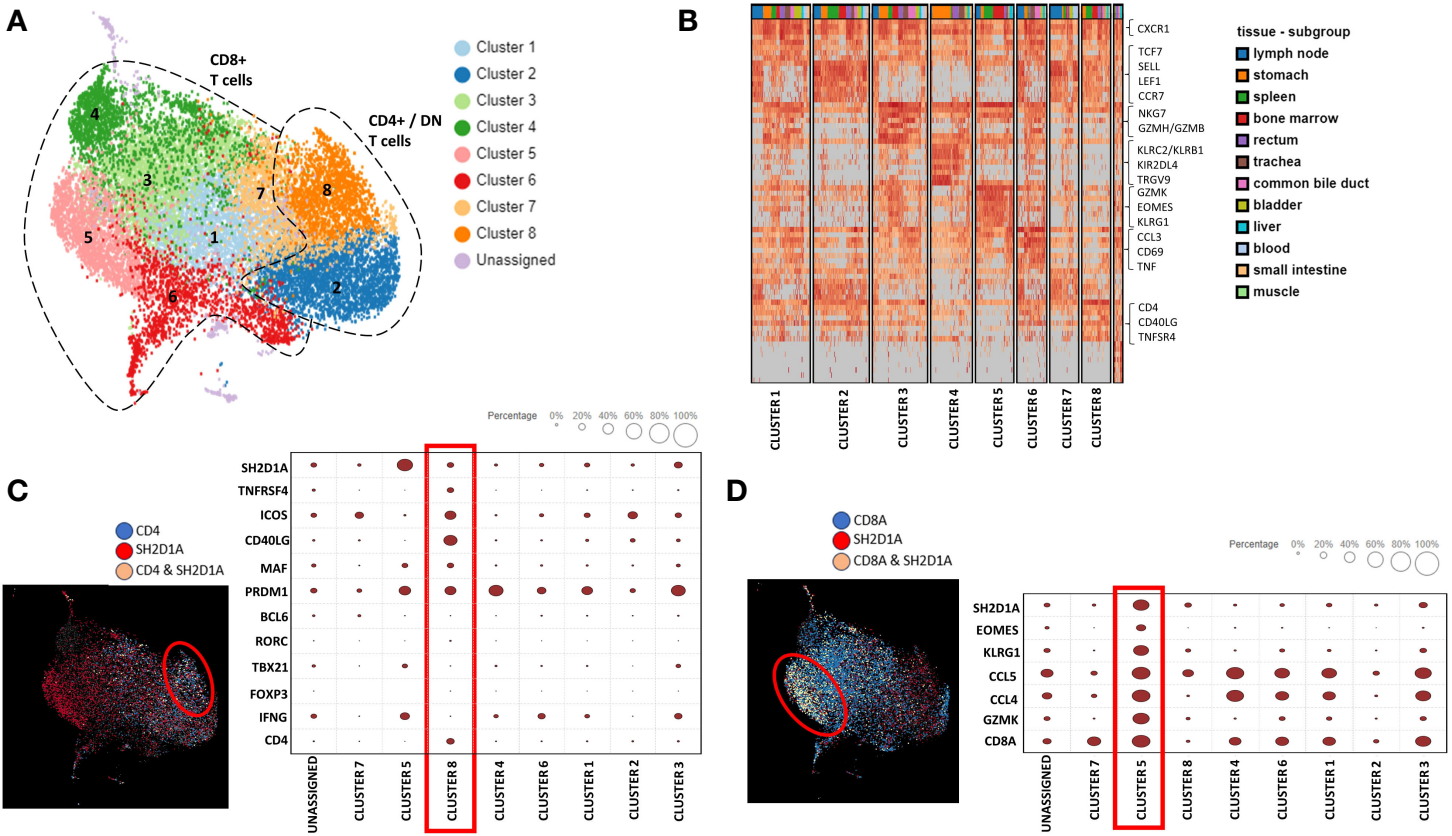
Single cell T cell transcriptome of major human organs confirms two distinct populations of SAP-high T cells, the TFH/TPH-like CD4 and GZMK+EOMES+ effector CD8. **(A)** Dimension reduction and clustering of 19,495 T cells was visualized using the UMAP algorithm. CD8+, CD4+ and double negative (DN) T cell clusters are shown. **(B)** Heat map analysis of gene expression in each T cell cluster. Columns represent selected differentially expressed signature genes in each cluster, and different clusters are exhibited in the rows. **(C)** Cellular composition of SAP gene (*SH2D1A*) and CD4 co-expression (left) mapped to cluster 8. Selected differentially expressed signature genes quantified (right). **(D)** Cellular composition of *SH2D1A* and CD8 co-expression (left) mapped to cluster 5 and selected differentially expressed signature genes quantified (right).

We next asked whether SAP-expressing T-cell clusters can be isolated from other major human organs. To answer this question, we used single-cell T cell RNA-seq data from biopsy samples of fifteen adult organs (20). Using this data, we identified 19,495 T cells, which we analyzed using unsupervised Louvain clustering and UMAP visualization (**Figure 7A**). We identified eight clusters of T cells, most of which were non-predominantly distributed amongst the different organs from which they were sampled (**Figure 7B**). Restricting the analysis to CD4 T cells, we found that the expression of the SAP gene, *SH2D1A*, localized predominantly to cluster 8. The additional genes enriched in cluster 8 included *ICOS* and *CD40-ligand* and transcription factors *MAF* and *PRDM1*, consistent with a TFH/TPH-like subset (**Figure 7C**).

Conversely, restricting the analysis to CD8 T cells, we found SAP expression in cluster 5. Enriched genes from this cluster included *EOMES, GZMK, KLRG1, CCL4*, and *CCL5* (**Figure 7D**). We were therefore able to confirm that organ infiltrating T cells, even in the absence of SLE, have two distinct populations of SAP-high T cells: the TFH/TPH-like CD4 and *EOMES* ^+^, *GZMK* ^+^ CD8 T subsets. These organ-infiltrating T cells isolated from a healthy volunteer suggest a baseline T cell presence that has the potential to become expanded in the context of SLE disease.

## Discussion

SLAMF receptors are important co-stimulatory TCR receptors critical for T-cell mediated B-cell development. In this work, we found elevated SAP levels in T cells from SLE patients compared to healthy controls. SAP levels correlated with markers of T cell activation, such as CD69 and HLA-DR expression. The highest levels of SAP expression in CD4 T cells were seen in the TPH, followed by the TFH, subsets.

TPH was first described in the synovium of patients with rheumatoid arthritis and has since been found to be expanded in systemic lupus, where its numbers correlate with disease activity and autoantibody titers (12, 13, 22). These cells were also identified in the kidneys of patients with lupus nephritis, where they correlated with pathogenic B cell subsets (23). In our SLE cohort, we found that SAP^+^TPH cells, but not total TPH cells, were associated with biopsy-proven LN. We believe that SAP^+^TPH cells identify a subset of functionally active TPH cells poised to support B cell maturation in the periphery more readily. SAP^+^TPH cells were also associated with active SLE (higher disease activity scores measured by SLEDAI-2K) and showed a trend towards an inverse correlation with complement C3 and C4 levels. The association between SAP^+^TPH cells and LN remained significant in multivariable analyses, controlling for common confounders such as age, sex, race, anti-dsDNA antibody levels, and complement levels. Additionally, using scRNA-seq data from kidney biopsy samples of patients with LN, we validated the presence of TPH-like T cells that express high levels of SAP mRNA and are further expanded in SLE compared to control.

The SAP gene (*SH2D1A*) is expressed on the X chromosome in humans. Loss of function SAP mutations result in disorganized germinal centers and lack of antigen-specific B cell differentiation, clinically manifesting as an X-linked immunodeficiency syndrome. Conversely, we propose that increased SLAM/SAP signaling functions augment T-B cell interactions and enhance autoantibody formation – conferring a risk for SLE. A single nucleotide polymorphism in the *SLAMF3* gene, which changes the conformation of the SLAMF3 receptor to have a more vital molecular interaction with SAP, is associated with the development of SLE (3, 24). Mutations in the SAP gene associated with SLE risk have also been reported, although the functional significance of these mutations is not well understood (25). On the other hand, the discovery of a *de-novo* frameshift mutation in the SAP gene of SLE-prone mice ameliorated the predisposition of these mice to develop SLE (11). Similarly, repression of SAP gene transcription by a transcription factor myocyte enhancer factor 2 (Mef2d) has been shown to inhibit SAP-dependent T-B synapse formation and prevent antigen-specific CD4 T cells from differentiation into germinal center TFH cells. *MEF2D* mRNA expression inversely correlates with TFH populations, autoantibodies, and SLEDAI scores in SLE (26). Our finding that SAP levels are increased in the circulation of SLE T cells, predominantly TFH and TPH cells, further supports the immunopathogenic role of SLAM-SAP signaling in SLE.

Our findings of increased SAP levels in SLE contrast with the work by Karampetsou et al., reporting decreased SAP in SLE T cells compared to controls (27). There are two possible explanations for this. First, we suspect there is clinical and molecular heterogeneity in SLE, but organ-specific SLE involvement is not considered by Karampetsou et al. Second, SAP levels in T cells are rapidly downregulated following T cell activation. Thus, differences in the processing/activation of PBMCs can lead to markedly different conclusions (28). Indeed, when Karampetsou et al. analyzed T cell lysates at different time points, the SLE’s decrease in T cell SAP levels was only seen after 5 hours of T cell culture treatment. Given that we found a correlation between SAP levels and markers of T cell activation (CD69, HLA-DR), it is possible that SAP level degradation over time in culture can account for the different observations in our study vs. Karampetsou et al.

Growing evidence suggests that tubulointerstitial inflammation strongly predicts long-term outcomes in lupus nephritis (29, 30). T cells compose more than 60% of all immune cells in the tubulointerstitium, and aggregates of T and B cells are seen in up to 50% of biopsy specimens (31, 32). It is believed that peripheral T and B cells traffic to the renal interstitium, where they become organized into tertiary lymphoid-like structures, promoting B cell isotype switching and *in-situ* auto-antigen responses. The expanded SAP^+^TPH cells in the circulation of patients with LN and the presence of these TPH-like cells in the kidney biopsy samples suggest a plausible pathological trajectory for these cells from the periphery to the lupus kidney. Furthermore, a possible target B cell population of TPH includes the CD11c^+^CD21^-^CXCR5^-^ age-associated B cells (ABC). This pro-inflammatory B cell subset rapidly expands and differentiates into plasmablasts upon activation (33). Indeed, the CD11c^+^ B cells in circulation correlate with markers of SLE disease activity and specifically with LN; the same cells are also found in the tissues of nephritic kidneys, where they localize to ectopic lymphoid follicles (34, 35). It is thus exciting to consider that the SAP^+^TPH subset identified by us to be associated with LN may represent a cognate partner to the ABC – with the possibility that both cell types can migrate into the target tissue, organize into ectopic germinal cells, and contribute to the development of LN.

**Figure 8:**
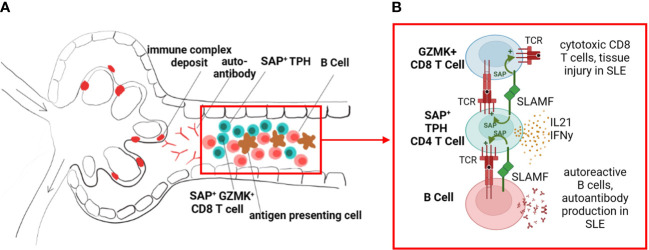
SLAM/SAP signaling is hypothesized to contribute to formation of tertiary lymphoid structures and *in-situ* development of autoreactive lymphocytes. **(A)** We propose that the expanded SAP-positive TPH cell population in SLE traffics to inflamed tissues, such as the kidney tubulointerstitial, and promotes B cell and effector CD8 T cell development *in situ*. **(B)** TPH cells support developing CD8 and B cells via direct cell-cell interactions, dependent on SLAMF-SLAMF binding and downstream SAP signaling, as well as release of cytokines such as interferon gamma (IFNy) and interleukin 21 (IL-21). The images were created with BioRender.com.

The differential gene analysis of SAP-high vs. SAP-low-expressing kidney infiltrating T cells confirmed that SAP expression was associated with members of the SLAMF family, specifically *SLAMF3* and *SLAMF7*. However, while the role of SAP is well described in the literature as being critical for CD4 T-B cell-mediated interactions, we additionally found that SAP expression was also strongly associated with a pro-inflammatory, CD8 effector T cell phenotype based on the high expression of *GZMK, EOMES*, *DTHD1*, and *CCL4* (MIP-1) and *CCL5* (RANTES). We found that the TPH-like CD4 subset and the *GZMK*+*EOMES*+ CD8 subset, expressing high levels of SAP, can be isolated from many healthy adult organs. Still, these cell populations were expanded in the kidneys of patients with lupus nephritis compared to control kidneys. The presence and function of the organ-infiltrating SAP-positive CD8 T cells in the steady state, and further expanded in inflammatory disease, still needs to be better understood. Given the gene enrichment of MHC-Class II antigen presentation genes in SAP-expressing T cells, it is possible that SLAM-SLAM signaling is not only critical for CD4 T-B cell signaling but also for *in-situ* CD4 T-cell assisted maturation of cognate CD8 T cells. Additionally, while there are multiple SLAM family receptors that share functional redundancy, the downstream signaling in the T cell converges on binding SAP. SAP then functions to recruit kinases that enhance phosphorylation of downstream TCR signaling proteins (i.e., ZAP-70, LAT) and thus T cell activation (8). Accelerated phosphorylation downstream of TCR enhances T cell hyper-responsiveness to self-ligands, suggesting increased SAP levels may have a mechanistic role in lowering the threshold of T-cell reactivity to self-antigen, thus initiating auto-reactive adaptive immunity (36). In this case, pharmacological disruption of the SAP signaling pathway may specifically target SLE’s more pathogenic helper and effector lymphocyte subsets (**Figure 8**).

Our work has several limitations. We studied a small number of patients, and future work will need to validate these findings in a larger sample. Additionally, while SLAM family receptor interactions are important in T-B cell signaling, SAP is expressed only in T and NK cells. The adaptor function of SAP is replaced by EAT-2 in B cells. Since our analysis focused on SAP expressing T cells, we cannot make any conclusions about B cell activation following SLAM family receptor activation.

Finally, we used the lupus kidney scRNA-seq data available from the AMP consortium. Still, given the relatively small number of T cells isolated from the kidney biopsy samples, we could not separately analyze CD4 from CD8 T cell clusters. Instead, we had to broaden the differential gene analyses to all T cells. As a result, compounded by the overall low-depth sequencing of the SAP gene, we tolerated a lower log2FC cutoff to increase sensitivity in discovering novel signaling molecules associated with high SAP expression. The analysis of the KEGG pathway in this context links the identified SAP-associated genes with Toll-like receptor signaling and cytosolic DNA-sensing responses, both of which are strongly linked to monogenic and polygenic SLE risk.

In summary, we have identified an expanded SAP^+^TPH population in the circulation and kidney samples of patients with biopsy-confirmed lupus nephritis. We believe this is a functionally active TPH cell population directly involved in *in situ* ectopic germinal center formations. We also identified a GZMK^+^ SAP^+^ CD8 T cell population with a pro-inflammatory cytokine and chemokine gene expression. The role of these T cell subsets in promoting direct tissue damage in lupus nephritis will be the subject of future research.

## Data availability statement

The original contributions presented in the study are included in the article/**Supplementary Materials**, further inquiries can be directed to the corresponding author/s. Sequencing data was obtained from publicly available datasets were analyzed in this study. This data can be found here: https://www.immport.org/shared/study/SDY997 and https://www.ncbi.nlm.nih.gov/geo/ under the accession number GSE159929.

## Ethics statement

The studies involving humans were approved by Columbia University Institutional Review Board. The studies were conducted in accordance with the local legislation and institutional requirements. The participants provided their written informed consent to participate in this study.

## Author contributions

YG: Conceptualization, Data curation, Formal analysis, Funding acquisition, Investigation, Methodology, Project administration, Visualization, Writing – original draft, Writing – review & editing. LG-P: Investigation, Resources, Writing – review & editing. LK: Conceptualization, Investigation, Resources, Writing – review & editing. SB: Data curation, Investigation, Methodology, Writing – review & editing. SL: Conceptualization, Investigation, Methodology, Writing – review & editing. RW: Conceptualization, Methodology, Supervision, Writing – review & editing. AA: Conceptualization, Funding acquisition, Methodology, Project administration, Resources, Supervision, Writing – review & editing. AM: Conceptualization, Funding acquisition, Methodology, Project administration, Resources, Supervision, Writing – review & editing.
