# Supplementary figures and images for: SAP-expressing T peripheral helper cells identify systemic lupus erythematosus patients with lupus nephritis
Source: Front Immunol. 2024 Mar 14;15:1327437. doi: 10.3389/fimmu.2024.1327437 (PMC10972949; doi:10.3389/fimmu.2024.1327437)

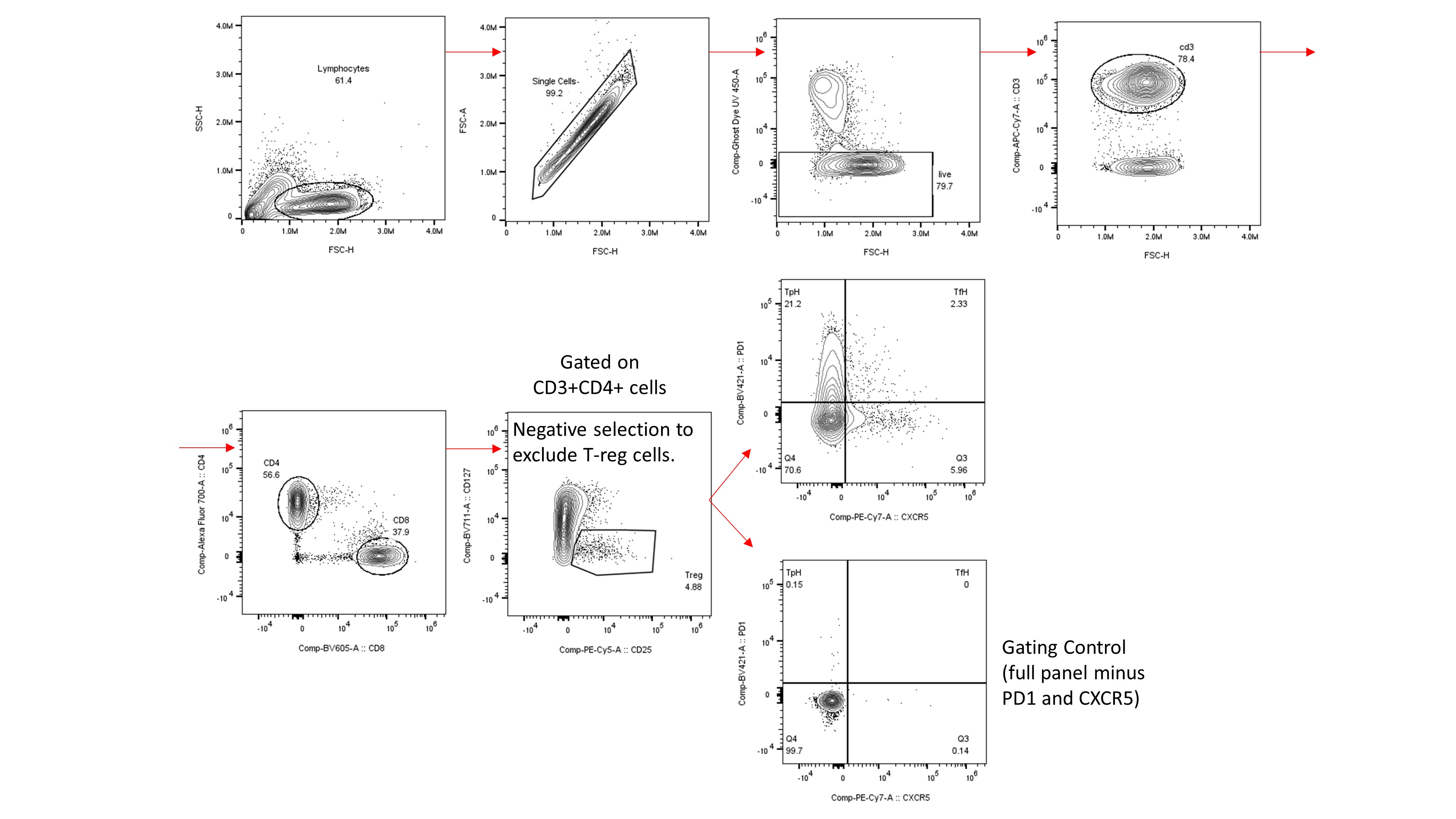

Supplement: Supplementary file 1 [file Image_1.jpeg]

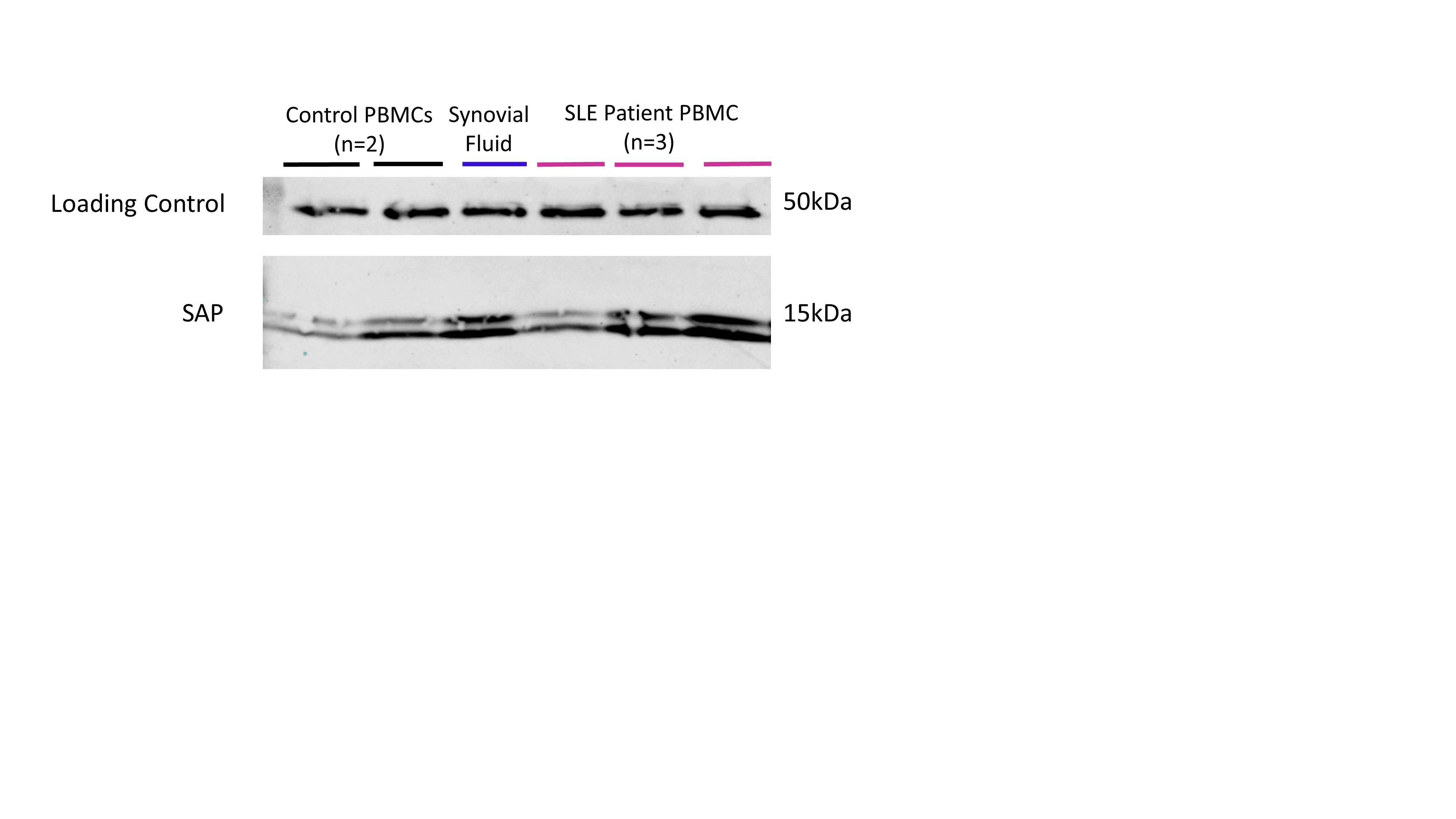

Supplement: Supplementary file 2 [file Image_2.jpeg]

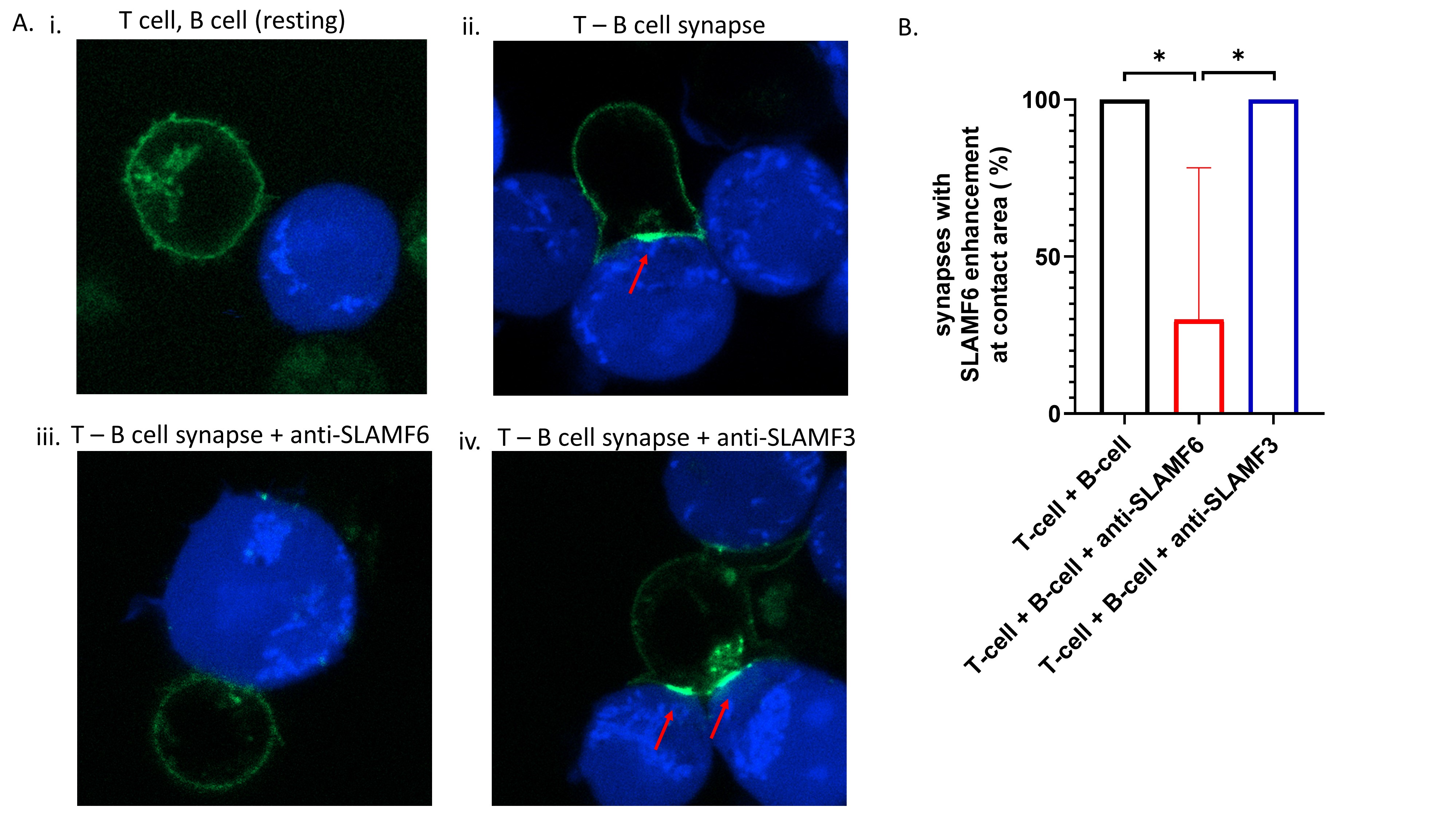

Supplement: Supplementary file 3 [file Image_3.jpeg]

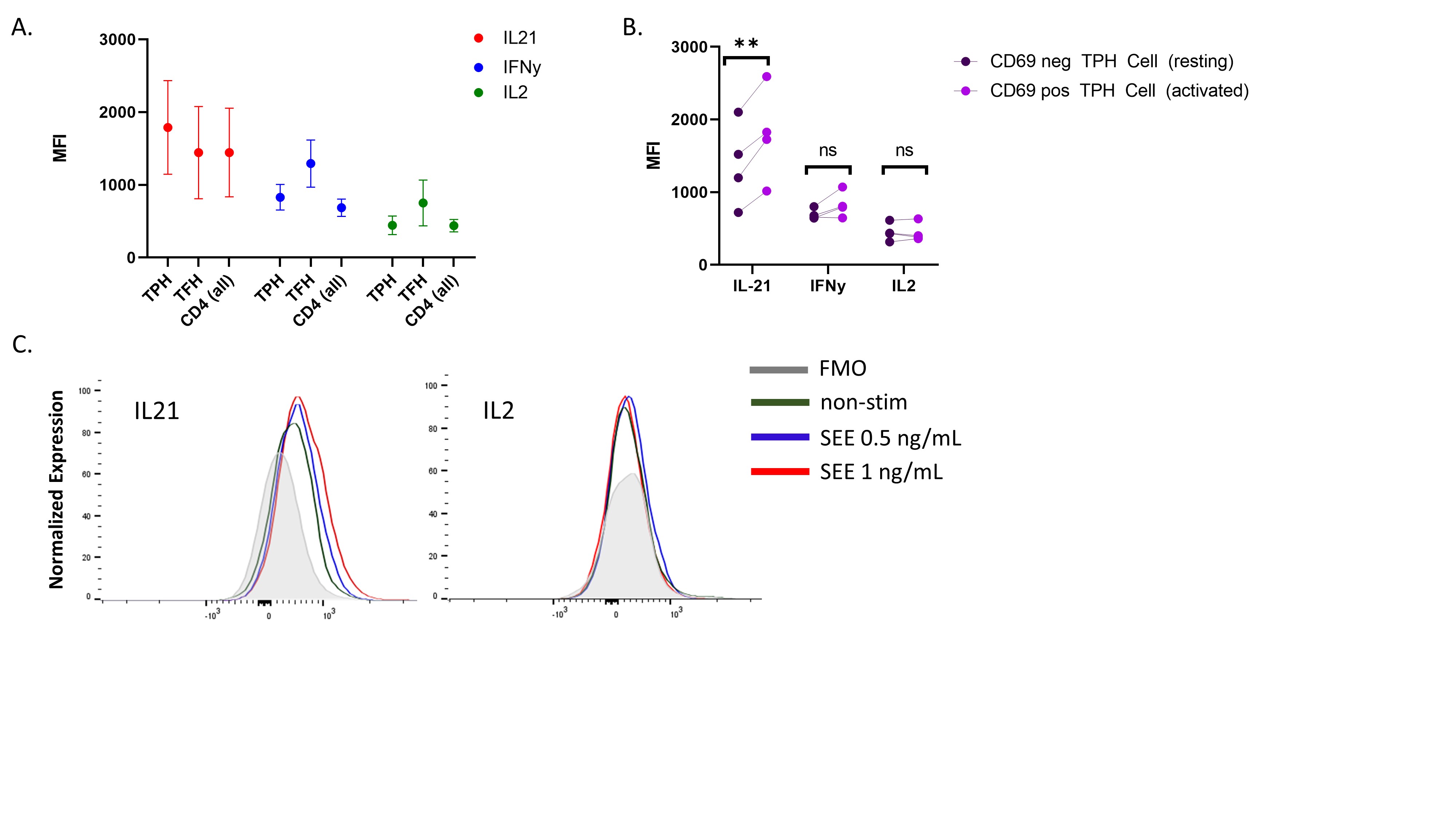

Supplement: Supplementary file 4 [file Image_4.jpeg]
