# Supplementary material for: SAP-expressing T peripheral helper cells identify systemic lupus erythematosus patients with lupus nephritis
Source: Front Immunol. 2024 Mar 14;15:1327437. doi: 10.3389/fimmu.2024.1327437 (PMC10972949; doi:10.3389/fimmu.2024.1327437)
Supplement: Supplementary file 5 [file Table_1.docx]

| Gene Name | logFC | P-value |
| --- | --- | --- |
| DTHD1 | 2.355551 | 2.97E-05 |
| FCER1G | 2.276613 | 0.004836 |
| GZMK | 2.035791 | 8.26E-11 |
| CRTAM | 1.381852 | 0.006452 |
| SH2D1A | 1.377981 | 3.95E-45 |
| HLA-DQB1 | 1.338356 | 0.027449 |
| EOMES | 1.203168 | 1.73E-05 |
| FCRL3 | 1.080756 | 0.002345 |
| CCL4 | 0.962647 | 8.30E-06 |
| NFX1 | 0.93218 | 0.025134 |
| PATL2 | 0.903657 | 0.043141 |
| SAMD3 | 0.884882 | 4.28E-05 |
| TTN | 0.866088 | 0.049656 |
| ATP8A1 | 0.848035 | 0.031628 |
| GZMA | 0.7834 | 1.89E-07 |
| CXCR4 | 0.783124 | 1.65E-07 |
| LY9 | 0.771538 | 0.012157 |
| SLAMF7 | 0.7524 | 0.001072 |
| CCL5 | 0.742392 | 4.71E-11 |
| GIMAP1 | 0.683942 | 0.00041 |
| CD63 | 0.67001 | 0.023547 |
| LINC00861 | 0.640585 | 0.034201 |
| HLA-DRB1 | 0.6313 | 0.010923 |
| CD74 | 0.603745 | 5.22E-16 |
| HLA-DPA1 | 0.600588 | 4.78E-05 |

Supplementary Table 1. List of genes upregulated in SAP high vs. low kidney infiltrating T cells.
